# Comparison of psyllium feeding at home and nasogastric intubation of psyllium and magnesium sulfate in the hospital as a treatment for naturally occurring colonic sand (geosediment) accumulations in horses: a retrospective study

**DOI:** 10.1186/s13028-016-0254-z

**Published:** 2016-10-12

**Authors:** Ritva Kaikkonen, Kati Niinistö, Tiina Lindholm, Marja Raekallio

**Affiliations:** 1 Oulu Equine Clinic (Previously Evidensia Equine Clinic Oulu), Äimänrautiontie 5, 90400 Oulu, Finland; 2 Department of Equine and Small Animal Medicine, Faculty of Veterinary Medicine, University of Helsinki, Koetilankuja 1, P. O. Box 57, 00014 Helsinki, Finland

## Abstract

**Background:**

Ingestion of geosediment (further referred as sand) may cause weight loss, diarrhea and acute or recurrent colic in horses. Our aim was to compare the efficacy of three treatment protocols in clearing colonic sand accumulations in clinical patients. This retrospective clinical study consisted of 1097 horses and ponies, which were radiographed for the presence of colonic sand. Horses included to the study (n = 246) were displaying areas of sand in the radiographs of ≥75 cm^2^ and were treated medically monitoring the response with radiographs. The horses were assigned into three groups based on the given treatment: Group 1 was fed psyllium [1 g/kg body weight (BW)] daily at home for a minimum of 10 days (n = 57); Group 2 was treated once with psyllium or magnesium sulfate by nasogastric tubing followed by daily feeding of psyllium (1 g/kg BW) at home for a minimum of 10 days (n = 19), and Group 3 was treated by daily nasogastric tubing for 3–7 days with psyllium and/or magnesium sulfate (1 g of each/kg BW) (n = 170).

**Results:**

The initial area of sand did not differ significantly between the treatments. Group 3 had significantly less residual sand than Groups 1 and 2, and the proportion of resolved horses was higher in Group 3 than in Groups 1 and 2.

**Conclusions:**

Daily nasogastric tubing with psyllium and/or magnesium sulfate for 3–7 days removes large accumulations of sand from the colon in horses more effectively than feeding psyllium for at least 10 days.

## Background

Ingestion of sand (geosediment) may cause serious, potentially life-threatening disease in horses living in areas with sandy soils or kept in sand paddocks. Colonic accumulation of geosediment, further referred as sand, is a geographically specific problem [1–3]. Sand accumulations in the gastrointestinal tract can result in poor performance, weight loss, chronic intermittent diarrhea, and acute, chronic or recurrent colic [3–5]. The methods used to diagnose sand accumulations include abdominal auscultation [6], rectal examination, the fecal glove test [7], ultrasonographic evaluation of the ventral colon [8], abdominal radiography [9–11], and laparotomy [12]. Radiography is also a useful means for monitoring the resolution of sand accumulations in clinical cases [3, 9, 13].

Abdominal radiography is the most reliable method for detection of an intestinal sand, since it provides an objective measurement of both the height and length of the sand, which cannot be evaluated with other methods such as abdominal ultrasonography [8] or abdominal auscultation [6]. Horses with small sand accumulations can be asymptomatic. For example, in a radiographic study horses with colic attributed to sand had larger sand accumulations (median height 9 × length 26.5 cm) than asymptomatic control horses (median 0.9 × 8.3 cm) [10]. Similarly, a significant relationship between acute colic and the size of the sand accumulation has been demonstrated [4].

Several treatments have been used to medically manage sand accumulations in horses, but their efficacies are largely unknown. The medical treatments used for clinical cases and in some experimental studies include nasogastric intubation and feeding of psyllium mucilloid (hereafter psyllium) from the desert Indian wheat *Plantago ovata* [13–16], magnesium sulfate (MgSO_4_) [9, 13], dioctyl sodium succinate, mineral oil, and their combinations [3, 9, 13, 17, 18].

Although published data are available on the effects of psyllium on evacuation of sand from the gastrointestinal tract [14], studies directly comparing psyllium feeding [16, 19–21] with administration by nasogastric intubation [13, 15, 19] are lacking. Moreover, the outcomes of previous studies are contradictory. No benefit of administration of psyllium, either fed or administered via nasogastric intubation, could be demonstrated for removal of sand after surgically placement of sand in the caecum in one study [19]. In contrast, a combination of MgSO_4_ 1 g/kg body weight (BW) and psyllium 1 g/kg BW administered via nasogastric intubation once per day for 4 days was significantly more efficient than the same doses of psyllium or MgSO_4_ alone for eliminating sand accumulations in another study [13]. A control group consuming sufficient hay cleared sand more efficiently than horses treated with psyllium in a study evaluating experimentally induced sand accumulations [21]. A further study fed horses sand and then treated them with mineral oil alone or combined with psyllium (1 g/kg BW twice per day) [15]. The mineral oil was administered by nasogastric intubation, while the psyllium was fed. Mineral oil combined with psyllium was more efficient at evacuating sand from the intestine than mineral oil alone.

In the authors’ geographical area in Finland, many owners feed psyllium periodically as a prophylaxis to horses kept in sand paddocks. Psyllium is reasonably priced, and many horses consume it either mixed with water, or water and concentrate. There are various types of psyllium supplements on the market: whole seed, granules, and a powdered form. The recommended dose of psyllium for sand removal varies from 0.5 to 1 g/kg [18], although the manufacturers’ dose recommendations for sand removal are often substantially lower. To our knowledge, all the previous psyllium feeding studies were conducted in experimental horses in controlled environments [15, 16, 19].

The aim of this retrospective study was to assess whether daily nasogastric intubation of psyllium, MgSO_4_, or both would be more effective than feeding psyllium at home to horses for the treatment of naturally occurring colonic sand accumulations in a population of clinical patients. The study also evaluated whether single nasogastric intubation followed by feeding psyllium at home is beneficial for sand removal. The efficacies of the treatments were evaluated by comparing the areas of the sand accumulation in the abdominal radiographs before and after treatment.

## Methods

Records of horses treated in the Helsinki University Veterinary Teaching Hospital (Clinic A) and the Evidensia Equine Clinic Oulu (Clinic B) between 1 January 2009 and 31 December 2014 were reviewed in this retrospective study. A total of 1097 horses and ponies had abdominal radiographs taken during this period. The most common reasons for the radiography were colic, loose feces, poor performance, or the owner’s wish, based on the horse’s altered behavior in animals with known access to sand. The horses were included in the study if they fulfilled the following criteria: an area on the radiograph consistent with the presence of sand ≥75 cm^2^, no abdominal surgery performed before treatment, horses treated medically either at home or in the hospital, and follow-up radiographs available. The horses treated at home had to be re-radiographed within 40 days, and those treated in the hospital within 8 days from initiation of the treatment. The sand accumulation was categorized as resolved if the area of sand accumulation was <25 cm^2^ in control radiographs [13]. If the same horse was treated again later, only the first treatment period was included in the study. All included horses were fed hay, haylage or a grass based diet.

The cranioventral abdomen of the horse was radiographed, using a right-to-left-standing lateral view, and the cassette placed in a ceiling-mounted wall stand or hanging cassette holder with a grid. The horses were sedated with intravenous detomidine 10 μg/kg if needed for radiography. Regular x-ray cassettes were used (Fujifilm IP cassette, type C, size 35.4 × 43 cm; Fujifilm, Tokyo, Japan). The distance between the horse and the machine was approximately 1.5 m. The radiographs were taken with a digitalized device (Shimadzu UD150B-40/Shimadzu MUX-10; Shimadzu Corp., Kyoto, Japan) and viewed with a commercial software program (Jivex, VISUS Technology Transfer, Palo Alto, CA, USA); the exposures were a maximum of 131 kV and 80 mAs. The edges of the sand accumulation were outlined individually in each radiograph, and the area of sand in the two-dimensional radiograph was calculated, using a software package, as previously described [13]. The radiographs were evaluated by RK (Clinic B) or KN (Clinic A).

In all, 246 horses fulfilled the inclusion criteria. They were further sorted into groups according to the type of treatment protocol, as follows:

1. Psyllium (various commercial brands) fed at home (n = 57) dose 1 g/kg once daily for a minimum of 10 days.
2. Administration of psyllium and/or MgSO_4_ only once by nasogastric tubing, after which feeding of psyllium at home (n = 19) dose 1 g/kg once daily for a minimum of 10 days.
3. Hospital treatment: daily nasogastric tubing for 3–7 days with psyllium and/or MgSO_4_, both 1 g/kg (n = 170). There was some individualized dosing of medication [i.e. horses with very loose feces or high serum magnesium concentrations received 0.5 g/kg MgSO_4_ and/or only psyllium (n = 10), and some horses received only MgSO_4_ (n = 7)].

During their stay in the hospital, the horses were box-rested and hand-walked, or turned out into a concrete paddock with no access to sand. The owners of the horses were advised to prevent further sand ingestion at home, either by muzzling the horses or changing the feeding management and avoiding turnout in sand paddocks, if possible.

Since the data were not normally distributed, the differences between the treatments were analyzed with Kruskal–Wallis analysis of variance and the Mann–Whitney *U* test using statistical software (IBM SPSS Statistics 23, IBM Corp., Armonk, NY, USA). The outcome of the treatment was recorded as resolved if the area of sand in the follow-up radiograph was less than 25 cm^2^ [10]. Pearson’s Chi-squared test was used to compare the proportion of resolved horses between treatment groups 1, 2, and 3, and Fisher’s Exact Test for pairwise comparisons between the groups. A *P* value of <0.05 was considered statistically significant.

## Results

The main reasons for abdominal radiography being performed are listed in Table 1. The most common reason for abdominal radiography was acute colic, but there were various symptoms in the horses presented to clinics. In Clinic A most of the acute colics were treated with daily nasogastric tubing, whereas in Clinic B acute colics were often treated with one nasogastric tubing and further feeding of psyllium.

**Table 1:** Complaints for the horses included in the study and treated with feeding (Group 1), nasogastric tubing once and feeding (Group 2), or daily nasogastric tubing (Group 3) of psyllium and/or MgSO_4_

Reason for radiographs^a^	Group 1 (n = 57)	Group 2 (n = 19)	Group 3 (n = 170)	All (n = 246)
Colic: acute	12	17	104	133
Colic: recurrent	1	0	27	28
Loose feces	28	2	36	66
Poor performance	16	1	10	27
Weight loss	3	0	7	10
Owner suspected sand	3	0	12	15
Hyperesthesia, behavioural changes	0	0	11	11
Not known	2	1	7	10

^a^52 horses were presented with a history and complaint of more than one clinical symptom

The initial area of sand did not differ significantly between the groups (*P* = 0.103). Horses treated with daily nasogastric intubation had significantly less residual sand and the proportion of resolved horses was higher than that of horses fed psyllium at home (Table 2).

**Table 2:** Outcome of horses with colonic sand accumulations and treated with feeding (Group 1), nasogastric tubing once and feeding (Group 2), or daily nasogastric tubing (Group 3) of psyllium and/or MgSO_4_

	Group 1	Group 2	Group 3
Number of horses in Clinic A	11	2	164
Number of horses in Clinic B	46	17	6
Total number of horses	57	19	170
Sand before treatment, median (cm^2^)	227	456	285
95 % confidence interval for median (minimum–maximum)	176–332 (75–1205)	242–631 (76–1241)	226–314 (79–1021)
Sand after treatment, median (cm^2^)	103*	106^†^	6
95 % confidence interval for median (minimum–maximum)	64–152 (0–810)	32–385 (0–792)	0–38 (0–828)
Number of resolved horses (sand after treatment <25 cm^2^)	14/57*	4/19^†^	91/170

* Significant difference between Groups 1 and 3 at P < 0.05

^†^Significant difference between Groups 2 and 3 at P < 0.05

No significant differences were detected between Groups 1 and 2

In 65 horses (35 in Group 1, 2 in Group 2, and 28 in Group 3), the exact area of sand could not be measured at the beginning of the study, due to the large sizes of the accumulations. Therefore, the size of the accumulation was estimated. In the control radiographs, the exact area could not be measured, for similar reasons, in 10 horses in Group 1 and 7 in Group 3. For example, in one horse radiographed on several occasions within a short period of time, on day 1 the size of the accumulation was 156 cm^2^. The horse was fed psyllium at home, but it was observed eating sand, and on day 7, the size of the accumulation was 701 cm^2^.

Most horses (63 %) presented between October and December. Of nine horses from Groups 1 and 2 that were diagnosed and treated between December and February, three were able to clear the sand with psyllium feeding.

## Discussion

In the present study, repeated administration of psyllium and/or MgSO_4_ by daily nasogastric tubing in the hospital resolved the sand accumulations more effectively and probably in a shorter time period than did feeding psyllium at home. In this retrospective material, most of the horses treated with nasogastric tubing were administered both psyllium and MgSO_4_. The number of horses administered only psyllium or MgSO_4_ was too small to make any comparisons between these treatments. However, in a previous prospective study the authors have demonstrated that the combination of psyllium and magnesium sulphate is more effective than either of the compounds alone [13].

More than half of the horses treated with nasogastric tubing where considered resolved within eight days, whereas less than one-third of the horses fed psyllium at home had resolved the sand in the control radiographs taken within 40 days. Although keeping the horse in the hospital for daily nasogastric intubation is costly, it is not only more efficient, but could eventually be more cost-effective than feeding psyllium at home, because the need for repeated radiological examinations and the delayed recovery increases the costs, as well as the risk of developing colic due to the accumulated sand. A single nasogastric intubation of psyllium in addition to further feeding of psyllium at home did not result in any additional benefit, compared with psyllium feeding only. However, the number of horses in this group was small and therefore the approximated 95 % confidence interval for the population median was wide.

It has previously been demonstrated that radiography can be a useful tool for monitoring the removal of sand [9]. Instead of only measuring the area a grading system that takes both the size and radiodensity of the sand accumulation into account has also been reported [10, 11]. Due to the numerous radiographs, the authors relied solely on the surface area in the present study, although this may have resulted in loss of some of the information. The magnification effect did probably not result in clinically relevant bias, because it was minor compared to the magnitude of the reduction of sand when the accumulation was considered resolved. The effect appears in the abdominal radiographs when the accumulation is located on the same side in which the cassette is held, in contrast to the other side.

Even though the most commonly used treatment protocols differed between the university teaching hospital and the private practice, no significant differences were detected in the areas of sand between the groups at the beginning. In some horses, the exact areas of sand could not be measured, due to the large sizes of the accumulations, because the margins were not entirely seen in the radiographs. This was evident both in the initial and follow-up radiographs. However, these difficulties in determining the exact size of some very large sand accumulations did not interfere with the evaluation of the outcome.

In the present study, sand accumulation was diagnosed in horses suffering from acute colic as well as in horses with chronic problems, such as loose feces or water with feces, weight loss and poor performance, all of which have been associated with colonic sand accumulation [3–5]. Some owners also reported hypersensitivity in the flank area while grooming the horse, which has not been previously reported to the authors’ knowledge. One study reported that some horses experienced episodes of abdominal pain during the hospital treatment [13], but this could not reliably be evaluated in the present study. However, the risk of acute colic must be taken into account when the treatment options are considered, and it may be necessary to monitor the horse in the hospital setting. Furthermore, it has been suggested, based on their experience, that horses with diagnosed sand accumulations that do not respond to the initial treatment should be managed surgically, due to risk of concurrent gastrointestinal pathology [3].

In the present study, there was some variation in the treatments used, including the specific medications used, their doses and the duration of the treatment. When horses were treated in the hospital (Clinic A), the most commonly used protocol was daily nasogastric tubing and evaluation of the outcome by radiographs for the first time after 4 days. The aim of treatment was to remove the majority of the sand, however the treatment was often discontinued prior to this occurring and horses with very large sand accumulations were not routinely treated with nasogastric intubation for more than 7 days to avoid nasal irritation and loss of patient compliance. This same practice has been previously reported [13]. In Clinic B, feeding psyllium at home was the most often-used treatment and presentation at the clinic for follow-up radiographs was regular. Only Clinic A could provide regular 24-h monitoring of the patients, and therefore in Clinic B only selected cases with increased risk for severe colic were hospitalized. This may have caused some bias in the study’s findings.

Whether horses treated at home were exposed to additional sand during the treatment remains unknown. Even though owners were instructed to prevent access to sand, it is unclear if these recommendations were followed or successful. In contrast, the hospitalized horses had no further access to sand. There is only anecdotal information available on how rapidly sand accumulates in the colon but reoccurrence of sand accumulation within 6 weeks has been reported [9]. It was not possible to control the problem of horses continuing to eat sand in the present study due to its retrospective nature. However, the authors propose that it would also be difficult with a prospective study using a population of clinical patients living in their home stables. As such, the authors suggest that this study reflects the real situation in the populations of clinical cases.

Some of the horses treated at home were diagnosed and treated between December and February, at which time the ground was frozen and covered with snow in northern Finland, where these horses lived. The authors considered it unlikely that the horses had access to sand at the time yet only three of these nine horses were able to clear the sand with psyllium feeding. In some patients, the radiographic area actually increased in size during the treatment period, even though there was apparently no access to sand. Most likely, sand from other parts of the colon was moving along and increasing the size of the accumulation as previously reported [9].

The length of the treatment at home varied, and many horses continued to consume psyllium for several weeks, because sand accumulation persisted in the colon. The follow-up period for the present study was restricted to 40 days, but clinical follow-up continued for those horses in which sand accumulation did not resolve. There has been some concern that feeding psyllium for long periods could decrease its efficacy, since the colonic flora may become able to metabolize it in increasing amounts [22]. In the present study, the owners were advised to temporarily halt the feeding after 4 weeks of treatment.

The impact of possible changes in diet on the outcome could not be evaluated in the present study. It has been suggested that having enough hay (minimum 1.5 % body weight) in the diet would enhance sand clearance [21]. Nevertheless, the horses in the present study were already fed a hay-based diet before the first radiographs, and yet they accumulated significant amounts of sand.

In the present study the occurrence of sand colic and other sand-associated diseases varied seasonally with most cases clustered at the end of the year suggesting that the most problematic time in our geographical region is after the pasture season but before the ground either freezes or becomes covered with snow. The thermal beginning of winter has varied over the years, which may explain the continuum of cases in some years until January. Weather conditions may be associated with sand eating but previous reports have come from different geographical areas than the present study [23].

## Conclusions

This retrospective study suggests that daily nasogastric tubing in hospital with MgSO_4_ and/or psyllium for 3–7 days removes sand accumulations from the colon in horses more effectively than feeding psyllium at home for at least 10 days.
